# A Fast and Efficient Hydrogen Chloride Sensor Based on a Polymer Composite Film Using a Novel Schiff-Based Triphenylamine Molecule as the Probe

**DOI:** 10.3390/ma18102291

**Published:** 2025-05-15

**Authors:** Hao Lv, Yaning Guo, Yinfeng Han, Jiaxin Ye, Jian Xiao, Xiaobing Hu

**Affiliations:** 1 College of Chemistry and Chemical Engineering, Baoji University of Arts and Sciences, Baoji 721013, China; 2 Shaanxi Key Laboratory of Phytochemistry, Baoji 721000, China

**Keywords:** triphenylamine derivatives, hydrogen chloride molecular probe, composite film

## Abstract

Hydrogen chloride (HCl) is one of the most hazardous air pollutants and can cause significant damage to human health and the environment. Therefore, the continuous quantitative monitoring of HCl is of great practical importance. In this work, a novel triphenylamine derivative, named TPTc-DBD, with a Schiff base structure was synthesized. The molecular structure of TPTc-DBD was determined by NMR analysis, FTIR analysis and single crystal diffraction analysis. On this basis, a porous polyvinylidene fluoride (PVDF) film containing TPTc-DBD was then prepared by a spin-coating method, and its sensitivity to HCl was evaluated by naked eye and ultraviolet-visible absorption spectrum, respectively. The detection limit of the composite porous film for HCl molecules was determined to be 5.8 mg/m^3^. Interestingly, the composite films absorbing HCl can be reactivated by NH_3_, which provides a cycle detection ability for HCl. After five testing cycles, the detection error remained below 1%. Furthermore, the microstructure of the film remained unchanged, highlighting its exceptional detection performance for HCl.

## 1. Introduction

Hydrogen chloride (HCl) is one of the most harmful atmospheric pollutants [1], and its excessive emission is harmful to both the human body and the environment. Therefore, its concentration in the workplace must be strictly controlled [2]. In recent years, with the progress of environmental protection and the construction of intelligent factories, the rapid, real-time and quantitative detection of pollutants has become an urgent need for society [3]. Traditional methods for detecting HCl include the mercury thiocyanate method [4], amperometric method [5], solid-state chemical method [6], etc. These detection methods have the disadvantages of being time-consuming, requiring a large sample size and having low sensitivity, which are obviously unable to meet the needs of society.

As an alternative to traditional methods, photochemical methods for the detection of HCl have the advantages of high sensitivity, good selectivity and short response time [7]. Currently, photochemical methods for quantitative detection of pollutants mainly include fluorescence spectrometry [8] and ultraviolet-visible absorption spectrometry [9]. Visual colorimetry has the advantage of easy operation, but usually its test accuracy is low. Currently, visual colorimetry is usually used for the detection of solutions [10], and the quantitative detection of HCl gas by this method has rarely been reported. Comparing these three methods, fluorescence detection and UV-Vis absorption detection are highly sensitive and accurate, while visual colorimetry is the simplest and lowest-cost method.

There are two kinds of molecular probe for detecting HCl gas. The first molecular probe is porphyrin derivatives [11], which have the disadvantages of low synthetic yield and high cost. The second molecular probe is Schiff base derivatives, which have the advantage of easy synthesis and low cost compared to porphyrin derivatives. While there is a risk of hydrolysis of Schiff base derivatives under acidic conditions, a common solution is to load Schiff base analogs onto organic polymers such as PVA [12] and PAM [13], but PVA and PAM are not very stable in highly acidic environment [14,15]. As an alternative, PVDF has excellent chemical stability in this environment. So, in this work, a novel triphenylamine derivative containing Schiff base structure named TPTc-DBD was first synthesized. Then, TPTc-DBD was used as a probe to prepare TPTc-DBD/PVDF composite film by spin-coating method. On this basis, the prepared composite film was used for the accurate monitoring of the concentration of HCl gas by means of visual colorimetry.

## 2. Materials and Methods

### 2.1. Materials

For this work, 4-Bromotriphenylamine, 5-bromo-2-thiophenecarboxaldehyde, 4-formylphenylboronic acid, tetrakis(triphenylphosphine)palladium, potassium carbonate, toluene, tetrahydrofuran, dichloromethane, petroleum ether, ethyl acetate, anhydrous ethanol and other reagents were purchased from Shanghai Taitan Chemical Co., Ltd. (Shanghai, China). and used directly without further purification.

### 2.2. Synthesis of Schiff-Based Triphenylamine Derivatives

#### 2.2.1. Synthesis of 5-(4-(Diphenylamino)phenyl)thiophene-2-carbaldehyde

**Figure 1:**
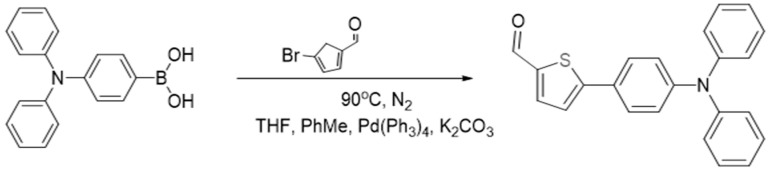
Synthesis scheme of TPTc.

The synthesis of 5-(4-(diphenylamino)phenyl)thiophene-2-carbaldehyde (TPTc) was carried out under the conditions of the Suzuki cross-coupling reaction [16,17,18] between 4-(Diphenylamino)benzeneboronic acid and 5-bromo-2-thiophenecarboxaldehyde according to the reaction scheme in Figure 1.

4-(Diphenylamino)benzeneboronic acid (1.73 mmol, 500 mg), 5-bromo-2-thiophenecarboxaldehyde (1.73 mmol, 331 mg), tetrakis(triphenylphosphine)palladium (0.17 mmol, 200 mg) and potassium carbonate (28.9 mmol, 4 g) were added to a round-bottomed flask containing 15 mL of toluene, 20 mL of tetrahydrofuran, and 10 mL of water, respectively. The reaction was stirred under nitrogen protection at 120 °C for 8 h. The progress of the reaction was monitored by thin-layer chromatography. At the end of the reaction, the reaction mixture was poured into 100 mL of water and extracted with dichloromethane. Subsequently, the organic layer was dried with anhydrous Na_2_SO_4_. The solvent was then evaporated, and the product was purified by silica gel column chromatography with petroleum ether/dichloromethane (15:1, *v*/*v*) as the eluent. This procedure resulted in the final product, yellow-green solid TPTc (519 mg, 61%). ^1^H NMR and ^13^C NMR spectra of TPTc were displayed in Figures S1 and S2.

M.P. 113.6 °C–115.3 °C. ^1^H NMR (400 MHz, CDCl_3_) δ 9.86 (s, 1H), 7.70 (d, J = 3.9 Hz, 1H), 7.52 (d, J = 8.6 Hz, 2H), 7.34–7.29 (m, 4H), 7.29–7.24 (m, 2H), 7.14 (d, J = 7.9 Hz, 4H), 7.10 (d, J = 7.6 Hz, 2H), 7.06 (d, J = 8.6 Hz, 2H).^13^C NMR (100 MHz, CDCl_3_) δ 182.51, 154.54, 149.12, 146.95, 141.32, 137.61, 129.44, 127.21, 126.13, 125.15, 123.84, 122.81, 122.34. HRMS (ESI+) was calculated for C_23_H_17_NOS, [M + H]^+^ 356.1112, giving 356.1105.

#### 2.2.2. Synthesis of N,N-Diphenyl-4,4′-biphenyldiamine

**Figure 2:**
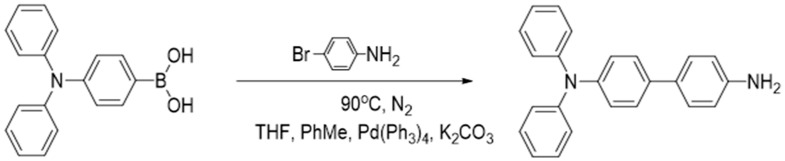
Synthesis scheme of DBD.

The synthesis of N,N-Diphenyl-4,4′-biphenyldiamine (DBD) was carried out by a Suzuki cross-coupling reaction between 4-(Diphenylamino)benzeneboronic acid and p-bromoaniline using a literature method [19]. The synthesis scheme of DBD was shown in Figure 2.

4-(Diphenylamino)benzeneboronic acid (1.73 mmol, 500 mg), p-bromoaniline (1.73 mmol, 298 mg), tetrakis(triphenylphosphine)palladium (0.17 mmol, 200 mg) and potassium carbonate (28.9 mmol, 4 g) were added into a round-bottom flask containing 15 mL of toluene, 20 mL of tetrahydrofuran and 10 mL of water. The reactant mixture was stirred under nitrogen protection at 120 °C for 8 h. The progress of the reaction was monitored using thin layer chromatography. At the end of the reaction, it was poured into 100 mL of water, extracted with dichloromethane and the organic layer was dried with anhydrous Na_2_SO_4_. After pouring out the solvent, it was then purified by silica gel column chromatography with petroleum ether/dichloromethane (5:1, *v*/*v*) as the eluent, and the final product was obtained as yellow waxy solid DBD (454 mg, 53%). ^1^H NMR and ^13^C NMR spectra of DBD were displayed in Figures S3 and S4.

M.P. 130.1 °C–134.8 °C. ^1^H NMR (400 MHz, CDCl_3_) δ 7.41 (t, J = 8.2 Hz, 4H), 7.26 (t, J = 7.8 Hz, 5H), 7.13 (s, 2H), 7.11 (dd, J = 6.0, 2.0 Hz, 4H), 7.01 (t, J = 7.3 Hz, 2H), 6.77 (d, J = 8.3 Hz, 2H), 3.99 (s, 2H). ^13^C NMR (100 MHz, CDCl_3_) δ 147.79, 147.79, 146.22, 146.22, 144.83, 144.83, 135.43, 135.43, 131.46, 129.15, 127.54, 126.99, 124.35, 124.09, 122.59, 115.66. HRMS (ESI+) was calculated for C_24_H_20_N_2_ [M + H]^+^ 337.1699, giving 337.1704.

#### 2.2.3. Synthesis of TPTc-DBD

**Figure 3:**
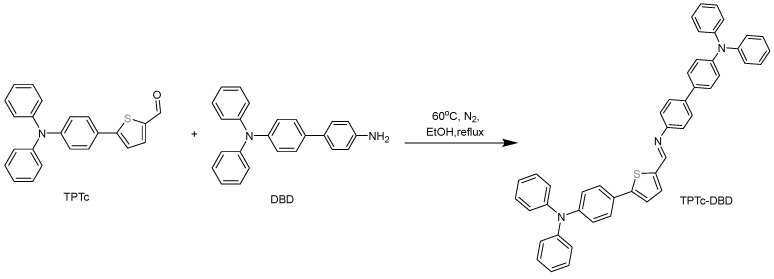
Synthesis scheme of TPTc-DBD.

The synthesis of TPTc-DBD was carried out by a Schiff base reaction using TPTc and DBD as the reactants, as shown in Figure 3. DBD (0.5 mmol, 168.08 mg) and TPTc (0.5 mmol, 177.55 mg) were added to a round bottom flask containing 10 mL of ethanol. After degassing, the solution was refluxed under nitrogen protection for 5 h. Afterwards, the reaction mixture was filtered, washed three times with 5 mL of hot ethanol and dried under vacuum. The target product TPTc-DBD was obtained (306 mg, 91%). ^1^H NMR and ^13^C NMR spectra of TPTc-DBD were displayed in Figures S5 and S6. The FTIR spectroscopy of TPTC-DBD was shown in Figure S6.

M.P. 183.4–184.7 °C. ^1^H NMR (400 MHz, CDCl_3_) δ 8.58 (s, 1H), 7.60 (d, J = 8.1 Hz, 2H), 7.54 (d, J = 8.5 Hz, 2H), 7.50 (d, J = 8.3 Hz, 2H), 7.35 (s, 2H), 7.31 (d, J = 7.8 Hz, 4H), 7.28–7.22 (m, 7H), 7.14 (d, J = 8.0 Hz, 10H), 7.11–7.05 (m, 5H), 7.03 (d, J = 7.1 Hz, 2H). ^13^C NMR (101 MHz, CDC_3_) δ 147.62, 138.55, 129.39, 129.24, 127.48, 127.27, 126.92, 126.36, 124.95, 124.40, 123.87, 123.57, 122.92, 121.49.

#### 2.2.4. Preparation of TPTc-DBD/PVDF Composite Porous Film

PVDF (3.5 g) was dissolved in 10 mL of DMF and ultrasonicated for three hours with stirring, and the resulting slurry was a homogeneous and transparent colorless liquid. In total, 5 wt% of TPTc-DBD was added to the DMF solution and continued to be ultrasonicated for one hour with stirring, until the slurry was a homogeneous and transparent light-yellow liquid. Afterwards, spin coating (400 r·min^−1^, 2 min) was carried out on a glass substrate using a rotary coater to obtain the films. The prepared film was removed from the spin coater and placed in a vacuum oven at 60 °C for 12 h. Then, the film was placed in anhydrous ethanol at 60 °C for 6 h. After removing it from the ethanol, the film was dried naturally to obtain TPTc-DBD/PVDF composite film. The brittle fracture of the film was performed after freezing with liquid nitrogen, and then the thickness of the film was observed by SEM analysis, which was about 4.378 μm (as shown in Figure S7).

#### 2.2.5. Preparation of the HCl Gas Detection Device

TPTc-DBD/PVDF composite porous film was placed on clean A4 paper, cut into strips of about 10 × 20 mm and then electrostatically adsorbed on slides. Then, a conical flask with a total volume of 145–160 mL was selected, and a layer of quartz sand and anhydrous calcium chloride mixture was spread on the bottom of the flask, which is about 50 mm thick, and then the above-mentioned slide was placed in the quartz sand with anhydrous calcium chloride mixture spread on the flask. The slides were placed in the conical flask with the quartz sand and anhydrous calcium chloride mixture, and the whole flask was heated to 140 °C in an oven for 30 min; the mouth of the flask was sealed with anhydrous calcium chloride-coated cotton wool, and then different volumes of HCl solution were added to the flask, and finally a beaker of the appropriate size was put on the lid, so as to make the whole flask into an airtight space, and the composite porous film was taken out after 5 min to test the ultraviolet absorption spectra. For desorption experiments, it is sufficient to replace the HCl solution in the above operation with concentrated ammonia.

### 2.3. Methods

FTIR spectra were measured at room temperature using a Perkin Amelmer IR spectrometer (Model: Thermo Scientific Nicolet iS50, Company: Thermo Fisher Scientific, Waltham, MA, USA). ^1^H NMR and ^13^C NMR spectra were measured using a Varian 400 MHz NMR (Model: INOVA 400MHz, Company: Agilent Technologies, Palo Alto, CA, USA) spectrometer with deuterated chloroform (CDCl_3_) as the solvent and tetramethylsilane (TMS) as the internal reference. Crystal structures were determined on a Bruker APEX II CCD (Model: Bruker Smart APEX II CCD diffractometer, Company: Bruker Corporation (Germany), Karlsruhe, Germany) area diffractometer equipped with graphite-monochromatized MoKα (λ = 0.071073 nm) using the φ–ω scan technique at different temperatures. The structure was solved by direct methods and refined on F2 by full-matrix least-squares methods using SHELX-97 [20,21,22]. High-resolution mass spectra (HRMS) were recorded on a Maxis instrument (Bruker Daltonics, Bremen, Germany) using electrospray ionization (ESI). The measurements were performed in a positive ion mode. Uv-Vis absorption spectra were recorded on a UV-2550 spectrometer with a wavelength range of 250–800 nm. Fluorescence spectra were measured using a Horiba Scientific Fluoro max-4 spectrofluorometer (FluoroMax-4, HORIBA Scientific, Edison, NJ, USA) at room temperature. The slit widths of excitation and emission measurements were both fixed at 5 nm. The fluorescence spectra of the compound and reference solutions were optimized by subtracting the solvent peak as part of the background. DFT computations were performed using the Gaussian 09 software package with the B3LYP functional and the 6-31G(d) basis set. The geometry of the molecule was optimized in the ground state using the DFT calculations with exchange-correlation functional PBE0 [23,24]. The culture of single crystals of the compound TPTc-DBD was carried out by a solvent volatilization method, using tetrahydrofuran as solvent.

## 3. Results and Discussion

### 3.1. Single Crystal Diffraction Analysis of TPTc-DBD

**Figure 4:**
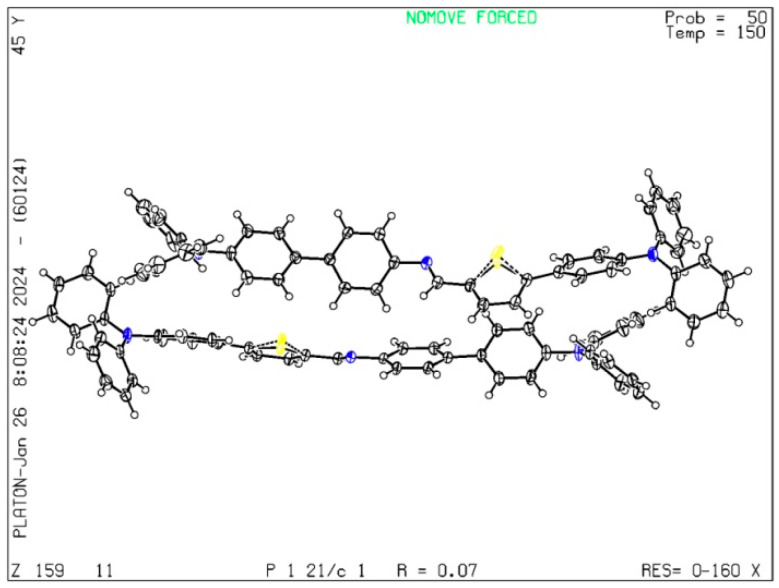
Crystal structure of TPTc-DBD. (The yellow small balls represent sulfur atoms, the blue small balls represent nitrogen atoms, the cross-shaped hollow balls represent carbon atoms, and the white small balls represent hydrogen atoms).

The single crystal diffraction pattern of the TPTc-DBD was measured and the result is shown in Figure 4. The detailed single crystal diffraction data of TPTc-DBD are shown in Table S1. The single crystal morphology of a TPTc-DBD molecule is columnar single crystal with the space group of monoclinic P21/C. There are no solvent molecules which appear in the crystal structure and the sulfur atoms are monoatomic disordered. The crystal structure of TPTc-DBD has been deposited at the Cambridge Crystallographic Data Centre (No. 2401114; deposit@ccdc.cam.ac.uk or http://www.ccdc.cam.ac.uk (accessed on 7 November 2024)).

### 3.2. Photophysical Properties of the TPTc-DBD and Its Selectivity to HCl and the Companion Gas

**Figure 5:**
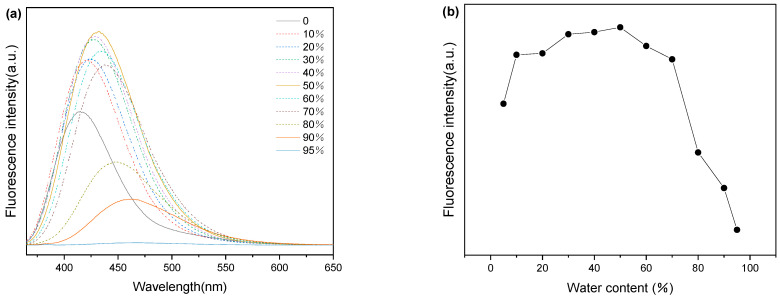
(**a**) Fluorescence spectra of TPTc-DBD with different water contents in THF/H_2_O (pH = 7.2). (**b**) The influence of water content on fluorescence intensity of TPTc-DBD in THF/H_2_O (pH = 7.2).

The TPTc-DBD molecule is a triphenylamine derivative, so its aggregation-induced luminescence (AIE) properties [25] were tested. The solution concentration of the TPTc-DBD was 10 μmol·L^−1^ during the study. The result is shown in Figure 5. The fluorescence intensity of the TPTc-DBD solution showed a trend of increasing and then decreasing with the increase in the volume fraction of water, and the emission wavelengths gradually red-shifted at the same time. The fluorescence intensity reached the maximum at the THF:H_2_O volume ratio of 50:50, with an emission wavelength of 432 nm, and the fluorescence quantum yield at this time was 43%. Furthermore, the fluorescence intensity decreased rapidly with the increase of H_2_O volume after the THF:H_2_O volume ratio reached 30:70, the fluorescence intensity was about half of the initial value when the THF:H_2_O volume ratio reached 10:90, and the emission wavelength was 461 nm. When the THF:H_2_O volume ratio reached 5:95, the fluorescence intensity was almost completely quenched. Overall, TPTc-DBD exhibits aggregation-caused quenching (ACQ) behavior, in which the aggregated state is non-fluorescent [26], and the compound exists as a yellow, solid powder at room temperature.

**Figure 6:**
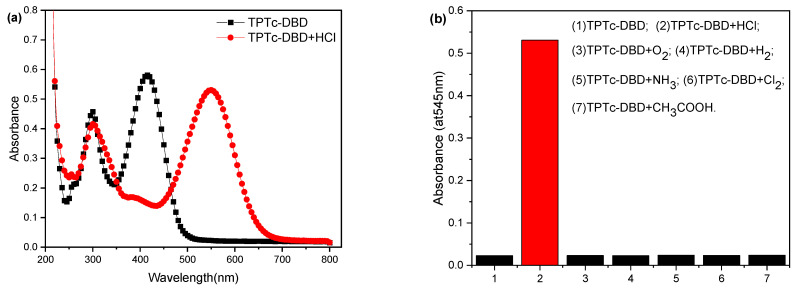
(**a**) Uv-Vis absorption spectra of TPTc-DBD (10 μmol·L^−1^) in acetonitrile solution, (**b**) response of TPTc-DBD (10 mol·L^−1^) to other companion gas.

In order to further verify the selectivity of TPTc-DBD molecules for HCl gas, the sensing performance of TPTc-DBD molecules for HCl in acetonitrile solution was tested, and the results are shown in Figure 6. The maximum UV absorption peak of TPTc-DBD was 415 nm, which red-shifted to 545 nm when HCl was added (Figure 6a). Moreover, the color of the solution changed from yellow to purple, which indicated that the TPTc-DBD molecule reacted with HCl. In order to investigate the selectivity of TPTc-DBD molecules to HCl, the effects of Cl_2_, NH_3_, O_2_ and H_2_, which are several HCl companion gases in chemical production, were also tested [27], as were CH_3_COOH vapors on the TPTc-DBD molecules for the detection of HCl, with a concentration of 10 μmol·L^−1^ of TPTc-DBD molecules, with a venting volume of 1 mL for both (Figure 6b). The results showed that the TPTc-DBD molecule has a good selectivity for HCl.

### 3.3. Morphological Study of Composite Porous Films of TPTc-DBD and PVDF

**Figure 7:**
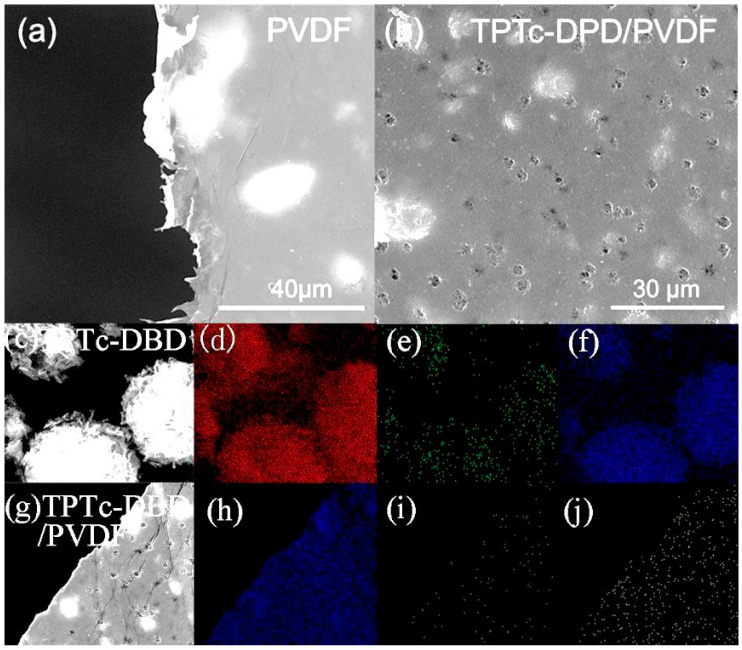
(**a**) SEM image of pure PVDF film; (**b**) SEM image of TPTc-DBD/PVDF composite film; (**c**) SEM image of TPTc-DBD powder; (**d**) C element distribution image of TPTc-DBD; (**e**) N element distribution image of TPTc-DBD; (**f**) S element distribution image of TPTc-DBD; (**g**) SEM image of TPTc-DBD/PVDF composite film; (**h**) F element distribution image of TPTc-DBD/PVDF composite film; (**i**) N element distribution image of TPTc-DBD/PVDF composite film; (**j**) S element distribution image of TPTc-DBD/PVDF composite film.

In order to achieve a simple and fast detection of HCl gas, composite films of TPTc-DBD and polyvinylidene difluoride (PVDF) were prepared in this study by spin coating method. Firstly, TPTc-DBD at 5% mass fraction was ultrasonically mixed with PVDF, and then the composite films were prepared by spin coating. After that, the films were rinsed with hot ethanol at 60 °C. On this basis, the composite film was subjected to SEM analysis (Figure 7). The results show that the pure PVDF film is a smooth film with some burr at the edge (Figure 7a). Many pores appeared on the TPTc-DBD/PVDF composite film, and the average diameter of these pores was about 3 μm (Figure 7b). The TPTc-DBD molecules showed a spherical distribution as shown in Figure 7c. The C element distribution, N element distribution and S element distribution images of TPTc-DBD were shown in Figure 7d, e and f, respectively, all of which showed a uniform distribution. The mapping analyses in Figure 7h–j showed that the three elements of C, F and S were relatively uniformly distributed in the composite film, indicating that the TPTc-DBD molecules were uniformly distributed within the PVDF matrix. The presence of pores is more favorable for the contact between HCl and TPTc-DBD molecules, suggesting a better HCl sensing performance on the fabricated composite films.

### 3.4. Sensing Performance of TPTc-DBD/PVDF Composite Film for HCl Gas

**Figure 8:**
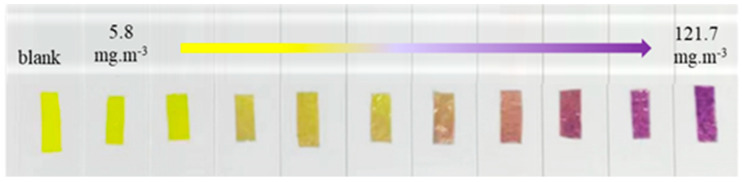
Photographs of TPTc-DBD/PVDF composite film under different concentrations of HCl atmosphere.

**Figure 9:**
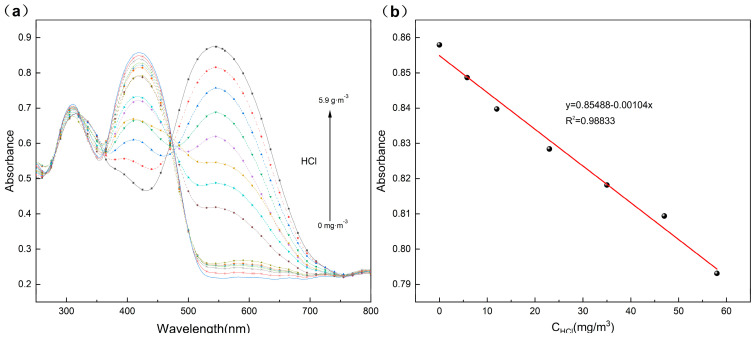
(**a**) Solid-state Uv-Vis absorption spectra of TPTc-DBD/PVDF composite film, (**b**) linear fitting curve of Uv-Vis absorbance at 417 nm of TPTc-DBD/PVDF composite film versus HCl concentration.

The porous composite films were placed in HCl gas with concentrations of 121.7 mg·m^−3^, 103.4 mg·m^−3^, 91.3 mg·m^−3^, 79.1 mg·m^−3^, 59 mg·m^−3^, 47 mg·m^−3^, 35 mg·m^−3^, 23 mg·m^−3^, 12 mg·m^−3^ and 5.8 mg·m^−3^, respectively, and the photographs were shown in Figure 8. The results showed that the color of the composite films deepened sequentially with the increase in HCl concentration, gradually changing from yellow to purple. The color of the composite film can remain unchanged at room temperature for several days. It is noteworthy that when the concentration of HCl gas increases, the intensity of the Uv-Vis absorption peak at 417 nm of the composite films gradually decreased, as shown in Figure 9a. These changes can be attributed to the variations in the energy band structure of TPTc-DBD. The molecular planarity of TPTc-DBD molecules increases and the degree of molecular conjugation is enhanced upon incorporation of HCl. The energy band gap calculated by Gaussian 09 B3LYP/6- 31G(d, p) of TPTc-DBD molecules decreases significantly from 2.943 eV to 1.173 eV when bound with HCl, as shown in Table 1.

There was a good linear relationship between the maximum absorbance values (at 417 nm) of the TPTc-DBD/PVDF composite films and the HCl gas concentration in the range of 5.8–59 mg·m^−3^, and the linear fitting equation was y = 0.85488 − 0.00104x (R^2^ = 0.98833) (Figure 9b). The limit of detection was determined by UV-Vis absorption spectrum as 3.56 ppm. Additionally, the theoretical limit of detection (LOD) can also be calculated using the following formula: LOD = 3ε/k, in which ε is the standard deviation of the absorbance intensity at 417 nm for 10 determinations of the blank solution, and k is the slope of the linear fit curve. So, the LOD of the composite film is calculated to be about 1.673 mg·m^−3^. TPTc-DBD/PVDF composite film is potentially a good sensing material for HCl. The sensing mechanism is clearly illustrated in Figure S8.

### 3.5. XPS Analysis of TPTc-DBD/PVDF Composite Film

**Figure 10:**
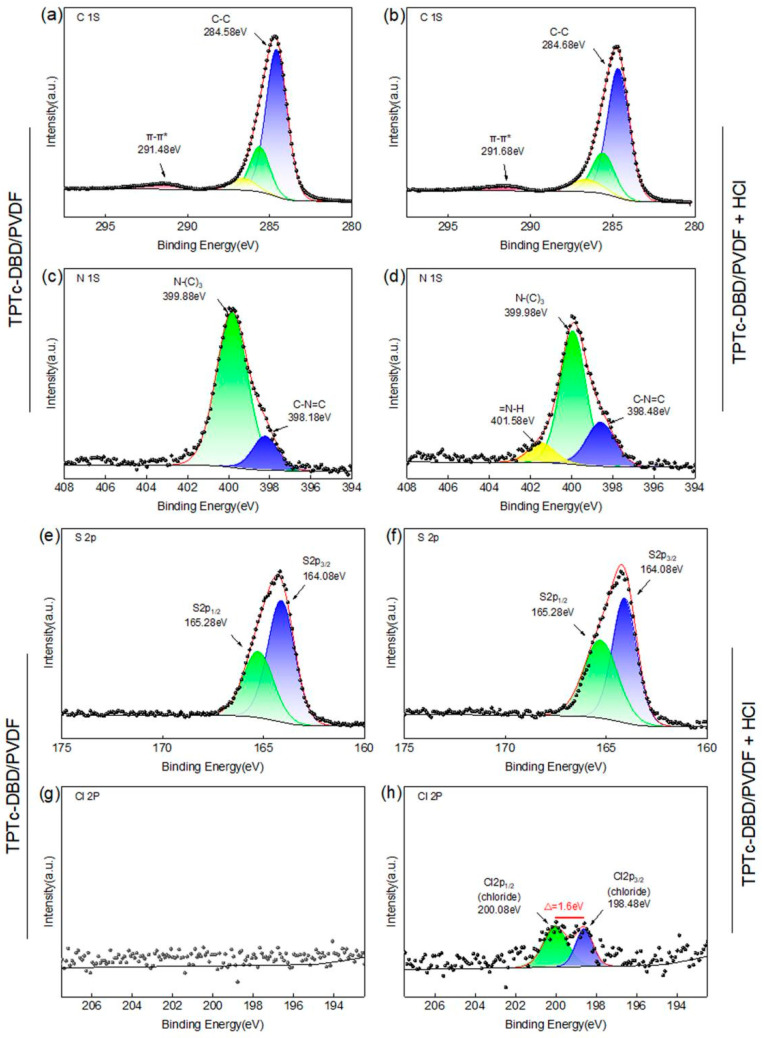
XPS spectra of C element (**a**) (**b**), N element (**c**) (**d**), S element (**e**) (**f**) and Cl element (**g**) (**h**) in TPTc-DBD/PVDF composite film before and after adsorption of HCl, respectively.

In order to investigate the binding morphology of the composite porous film with HCl, the electronic binding energy of each element of the composite film before and after the adsorption of HCl was characterized by XPS in this study (Figure 10). As can be seen from Figure 10a,b, the electronic binding energy spectra of element C before and after adsorption of HCl on the composite porous film did not change significantly, but the π-π* stacking peak at 291.68 eV was observed in both XPS spectra of element C, which explains the non-fluorescence of the aggregated state of the TPTc-DBD molecules. The electronic binding energy of element S did not change, suggesting that the HCl did not bind with the S atom in TPTc-DBD molecules (Figure 10e,f). While in Figure 10c,d, element N showed a new peak of =N-H at 401.58 eV, which indicated that the H atom of HCl was bound to the N atom in C=N on the TPTc-DBD molecules [28,29,30]. Before adsorption of HCl, no peak for element Cl was observed in the XPS spectrum of the composite porous film (Figure 10g). It is noteworthy that peaks at 200.08 eV and 198.48 eV were observed in the XPS spectrum of element Cl after adsorption of HCl on the composite porous film, and the difference between the two peaks was 1.6 eV (Figure 10h), which is typical of chloride peaks. This is a typical chloride peak. This indicates that the TPTc-DBD molecules on the composite porous film bind with HCl molecules to form a protonated Schiff base–chloride ion pair.

### 3.6. Theoretical Computational Study of HCl Binding to TPTc-DBD

The energy-optimized orbitals of TPTc-DBD and TPTc-DBD-H^+^ were calculated by Gaussian 09 B3LYP/6- 31G(d, p). The geometry of the molecules was optimized in the ground state using the DFT calculations with exchange-correlation functional PBE0 [20,21]. The results are shown in Table 1. The molecular planarity of TPTc-DBD molecules increases and the degree of molecular conjugation is enhanced upon incorporation of HCl. The energy band gap of TPTc-DBD molecules decreases significantly from 2.943 eV to 1.173 eV. The electron cloud in its HOMO orbitals is shifted towards the triphenylamine moiety, and the distribution of the electron cloud in the LUMO orbitals is shifted towards the Schiff base (-C=N) moiety. The theoretical calculations are consistent with the results of Uv-Vis absorption analysis.

### 3.7. The Cyclic Detection Performance of TPTc-DBD/PVDF Composite Film for HCl

**Figure 11:**
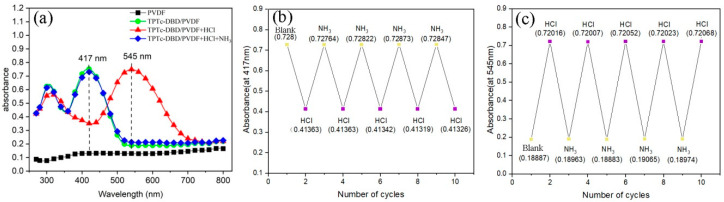
(**a**) UV-Vis absorption spectra of TPTc-DBD/PVDF composite films, (**b**) absorbance at 417 nm of TPTc-DBD/PVDF composite films in different atmospheric environments, (**c**) absorbance at 545 nm of TPTc-DBD/PVDF composite films in different atmospheric environments.

In order to investigate the cyclic detection performance of composite films for HCl gas, NH_3_ was selected as the desorption gas of TPTc-DBD/PVDF composite films after the adsorption of HCl. Hydrogen chloride (HCl) is an acidic gas, which can bind to the basic sites on the TPTc-DBD molecules in the TPTc-DBD/PVDF composite film, while ammonia (NH_3_) is an alkaline gas that can recapture the HCl from TPTc-DBD molecules. The Uv-Vis absorption spectra of pure TPTc-DBD/PVDF composite films, TPTc-DBD/PVDF composite films after adsorption of HCl and TPTc-DBD/PVDF composite films after adsorption of HCl and NH_3_ were tested separately. A total of five film samples were prepared for testing, one at a time. For each sample, the five cycles were performed on the same day with a 1-h interval between each cycle. The result showed that these samples exhibited good reproducibility. The results are shown in Figure 11. In Figure 11a, there is no obvious absorption peak in the range of 300–800 nm for the PVDF matrix. The TPTc-DBD/PVDF composite film exhibits two absorption peaks in the range of 300–800 nm, located at 320 nm and 417 nm, respectively, with the maximum absorption peak at 417 nm and an absorbance value of 0.728. When the TPTc-DBD/PVDF composite film adsorbs HCl, its absorbance value at 417 nm decreases from 0.728 to 0.414. As an alternative, it exhibits a distinct strong absorption peak at 545 nm, with an absorbance value of approximately 0.72, while the pure TPTc-DBD/PVDF composite film has an absorbance value of 0.189 at 545 nm. When the TPTc-DBD/PVDF composite film adsorbing HCl interacts with NH_3_ again, its UV-Vis absorption spectrum curve almost overlaps with that of the pure TPTc-DBD/PVDF composite film, demonstrating good recyclability. We repeated the process five times and recorded the absorbance values of the TPTc-DBD/PVDF composite film at 417nm (Figure 11b) and 545nm (Figure 11c), respectively. The results showed that after five testing cycles, the absorbance values of the composite film at 417 nm and 545 nm exhibited good repeatability, with a maximum error of only 0.94%. The test was carried out for ten cycles. The result is shown in Figure S9. The absorbance values of the composite film at 417 nm and 545 nm exhibited good repeatability after eight test cycles. Starting from the nineth test cycle, the absorbance values at 417 nm and 545 nm deviated to a certain extent, indicating that the stability of the film decreased to a certain extent at this time, and there was no significant change in the film morphology after ten test cycles (Figure S10). So, it can be seen that the TPTc-DBD/PVDF composite film has a good cyclic detection performance for HCl gas.

**Figure 12:**
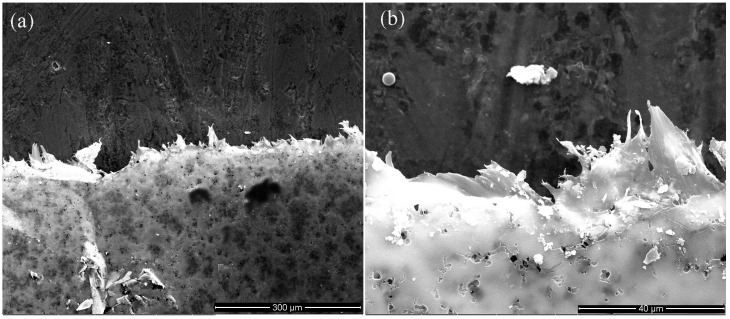
SEM of TPTc-DBD/PVDF composite films after five testing cycles, (**a**) ×400, (**b**) ×3000.

In order to further investigate the stability of TPTc-DBD/PVDF composite films, SEM analysis was conducted on the composite films before and after five testing cycles (Figure 12). Before testing, the composite film had a rich and uniform pore structure. After five testing cycles of HCl gas detection, it was clearly observed that the pore structure on the composite film remained intact. The composite porous film has good cycling stability.

## 4. Conclusions

In this study, a novel triphenylamine derivative TPTc-DBD with a Schiff base structure was first synthesized and its molecular structure was determined by nuclear magnetic resonance (NMR) and single crystal diffraction analysis. Then, TPTc-DBD was loaded onto polyvinylidene difluoride (PVDF) to prepare the TPTc-DBD/PVDF composite porous film. The film had a good detection performance for HCl, and its color changed gradually from bright yellow to dark purple with the increase in HCl concentration. The monitoring of HCl concentration can be realized under naked eye observation, and its minimum detection limit for HCl is about 5.8 mg/m^3^. Solid-state Uv-Vis absorption spectra analysis showed that, with the increase in HCl concentration, the intensity of the Uv-Vis absorption peak at 417 nm of the composite films gradually decreased. There was a good linear relationship between the maximum absorbance values (at 417 nm) of the TPTc-DBD/PVDF composite films and the HCl concentration in the range of 5.8–59 mg·m^−3^, and the linear fitting equation was y = 0.85488 − 0.00104x (R^2^ = 0.98833). XPS analysis revealed that the TPTc-DBD molecule on the composite porous film is bound to the HCl molecule rather than to the proton. Theoretical calculation displayed that the energy band gap of TPTc-DBD decreased significantly from 2.943 to 1.173 eV when combined with HCl molecules. Moreover, the TPTc-DBD/PVDF composite films can be reactivated by NH_3_ and regain its detection activity for HCl. This enables the cyclic detection of HCl.

## Figures and Tables

**Table 1:** Molecular frontline orbitals of TPTc-DDB and TPTc-DDB-H^+^.

	HOMO	LUMO	Eg/eV
TPTc-DBD	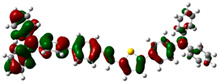	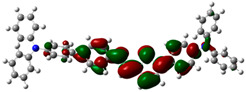	2.944 eV
TPTc-DBD-H^+^	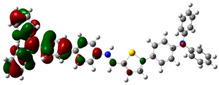	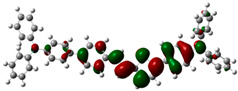	1.173 eV

## Data Availability

The original contributions presented in this study are included in the article/Supplementary Materials. Further inquiries can be directed at the corresponding author.

## Supplementary Materials

The following supporting information can be downloaded at: https://www.mdpi.com/article/10.3390/ma18102291/s1. Figure S1: ^1^H NMR spectrum of DBD; Figure S2: ^13^C NMR spectrum of DBD; Figure S3: ^1^H NMR spectrum of TPTC; Figure S4: ^13^C NMR spectrum of TPTC; Figure S5: ^1^H NMR spectrum of TPTC-DBD; Table S1: Single crystal diffraction data of TPTc-DBD molecules; Figure S6: Infrared spectroscopy of TPTC-DBD, DBD and TPTC; Figure S7: Cross-sectional structure of the TPTc-DBD/PVDF composite film; Figure S8: Schematic diagram of the thin composite film construction and its sensing mechanism to HCl; Figure S9: (a) Uv-Vis absorbance at 417 nm of TPTc-DBD/PVDF composite films in HCl and NH3 atmospheric environments during ten test cycles, (b) Uv-Vis absorbance at 545 nm of TPTc-DBD/PVDF composite films in HCl and NH3 atmospheric environments during ten test cycles; Figure S10: The morphology of TPTc-DBD/PVDF composite film after ten test cycles; Table S2: A comparison of sensing performance on HCl between some previously reported sensors and the sensor in this work. References [7,11,31,32,33,34,35,36,37,38,39] are cited in the supplementary materials.

## Abbreviations

The following abbreviations are used in this manuscript:

HCl	hydrogen chloride
PVDF	polyvinylidene fluoride
PVA	polyvinyl alcohol
PAM	Polyacrylamide
THF	tetrahydrofuran
TPTc	5-(4-(diphenylamino)phenyl)thiophene-2-carbaldehyde
DBD	N,N-Diphenyl-4,4′-biphenyldiamine
NMR	Nuclear Magnetic Resonance
M.P.	melting point
HRMS	high resolution mass spectrometry
DFT	density functional theory
CCDC	the cambridge crystallographic data centre
SEM	scanning electron microscopy analysis
HOMO	Highest Occupied Molecular Orbital
LUMO	Lowest Unoccupied Molecular Orbital
FTIR	Fourier transform infrared spectroscopy
