# Supplementary material for: A Fast and Efficient Hydrogen Chloride Sensor Based on a Polymer Composite Film Using a Novel Schiff-Based Triphenylamine Molecule as the Probe
Source: Materials (Basel). 2025 May 15;18(10):2291. doi: 10.3390/ma18102291 (PMC12113228; doi:10.3390/ma18102291)
Supplement: Supplementary file 1 [file materials-18-02291-s001.zip › materials-3593216-supplementary.pdf]

materials

MDPI

Supporting Information

A fast and efficient hydrogen chloride sensor based on a polymer composite film using a novel Schiff-based triphenylamine molecule as the probe

Hao Lv [1], Yaning Guo [1], Yinfeng Han [1], Jiaxin Ye [1], Jian Xiao [*,1,2] and Xiaobing Hu [*,1,2]

1. College of Chemistry and Chemical Engineering, Baoji University of Arts and Sciences, Baoji Shaanxi, People's Republic of China
2. [2] Shaanxi Key Laboratory of Phytochemistry, Baoji Shaanxi, People's Republic of China

* Correspondence: xjxs163@126.com (Jian Xiao); hxb.0917@stu.xjtu.edu.cn (Xiaobing Hu)

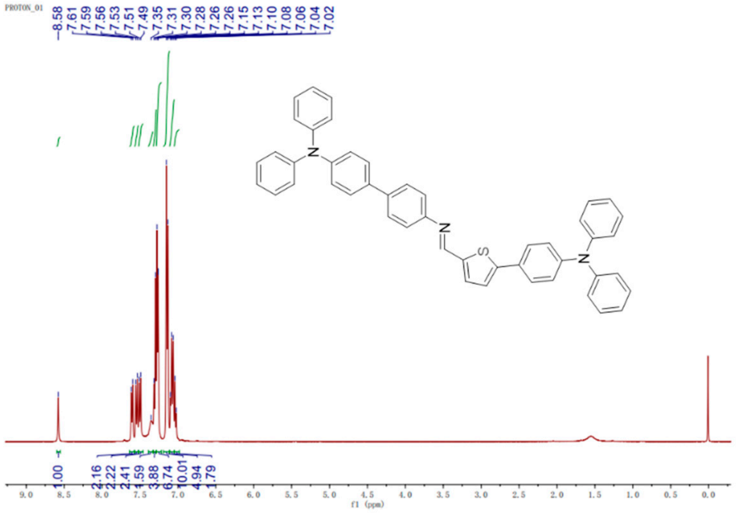

Figure S1. ^{1}H NMR spectrum of **TPTC-DBD.**

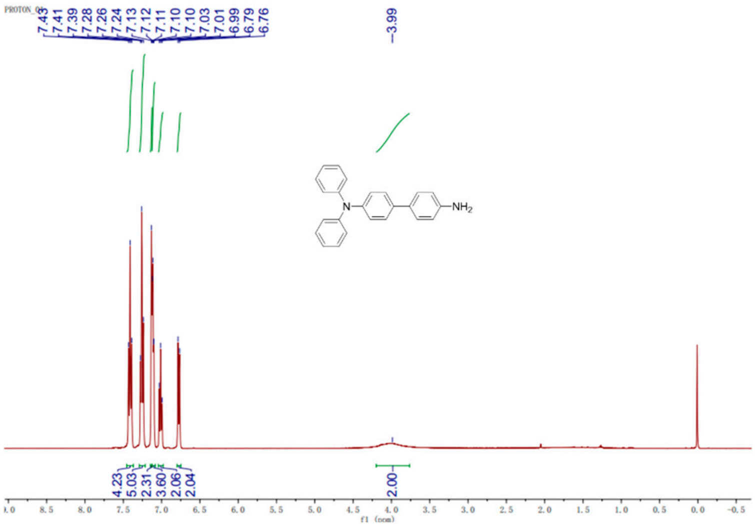

Figure S2. ^{1}H NMR spectrum of **DBD.**

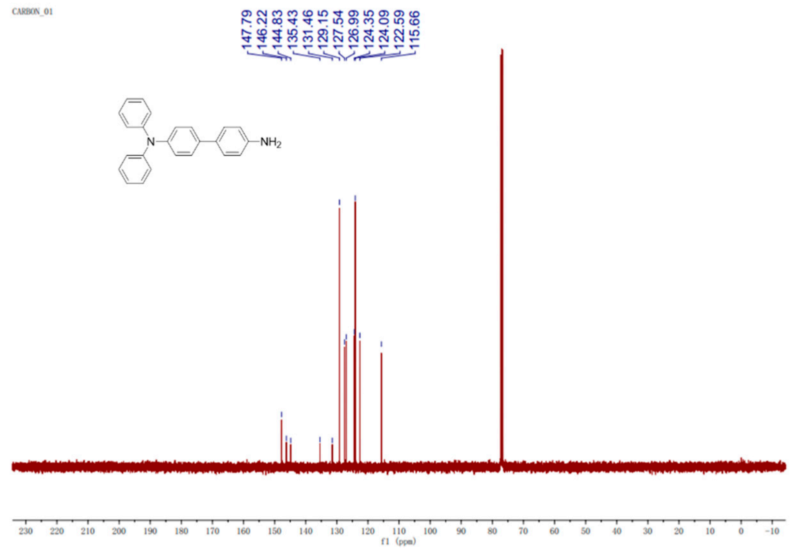

Figure S3. ^{13}C NMR spectrum of **DBD.**

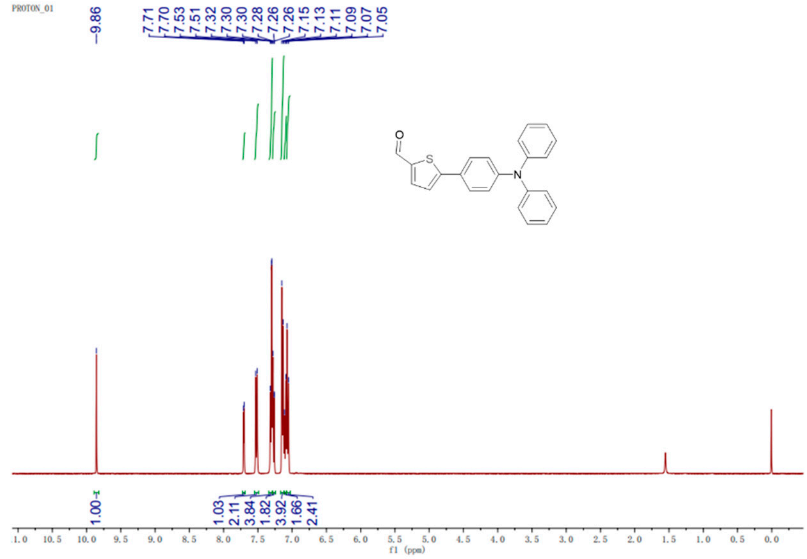

Figure S4. ^{1}H NMR spectrum of **TPTC.**

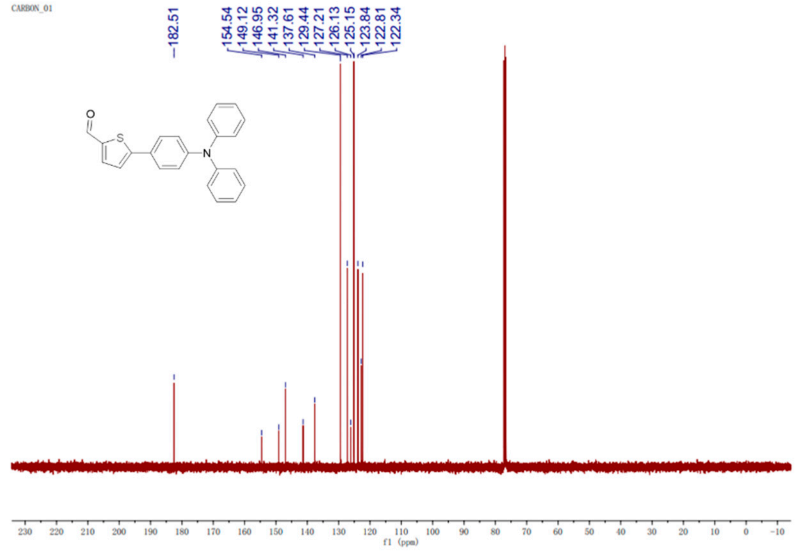

Figure S5. ^{13}C NMR spectrum of **TPTC.**

Table S1. Single crystal diffraction data of **TPTc DBD** molecules.

**Band precision:**	**C-C = 0.0042 A**		**Wavelength=0.71073**
Cell:	a=45.441(6)	b=9.2681(12)	c=17.117(2)
	alpha=90	beta=92.949(5）	gamma=90
Temperature:	150K		
	Calculated	Reported	
Volume	7199.3(16)	7199.2(16)	
Space group	P 21/c	P 1 21/c 1	
Hall group	- P 2ybc	-P 2ybc	
Moiety formula	C47 H35 N3 S	C47 H35 N3 S	
Sum formula	C47 H35 N3 S	C47 H35 N3 S	
Mr	673.84	673.84	
Dx, g cm-3	1 .243	1 .243	
Z	8	8	
Mu (mm-1)	0.128	0.128	
F000	2832.0	2832.0	
F000'	2833.94		
h,k,lmax	56,11,21	56,11,21	
Nref	14798	14638	
Tmin,Tmax	0.982,0.987	0.552,0.751	
Tmin'	0.98		
Correction method= # Reported T Limits: Tmin=0.552 Tmax=0.751			
AbsCorr = MULTI-SCAN			
Data completeness= 0.989		Theta(max)= 26.412	
R(reflections)=0.0682(11236)			wR2(reflections)= 0.1816(14638)
S = 1.063		Npar= 938	

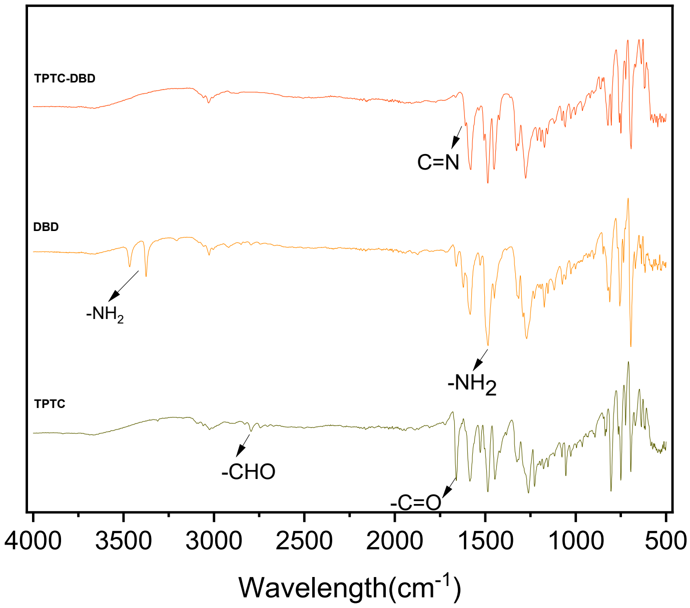

Figure S6. Infrared spectroscopy of **TPTC-DBD**、**DBD** and **TPTC.**

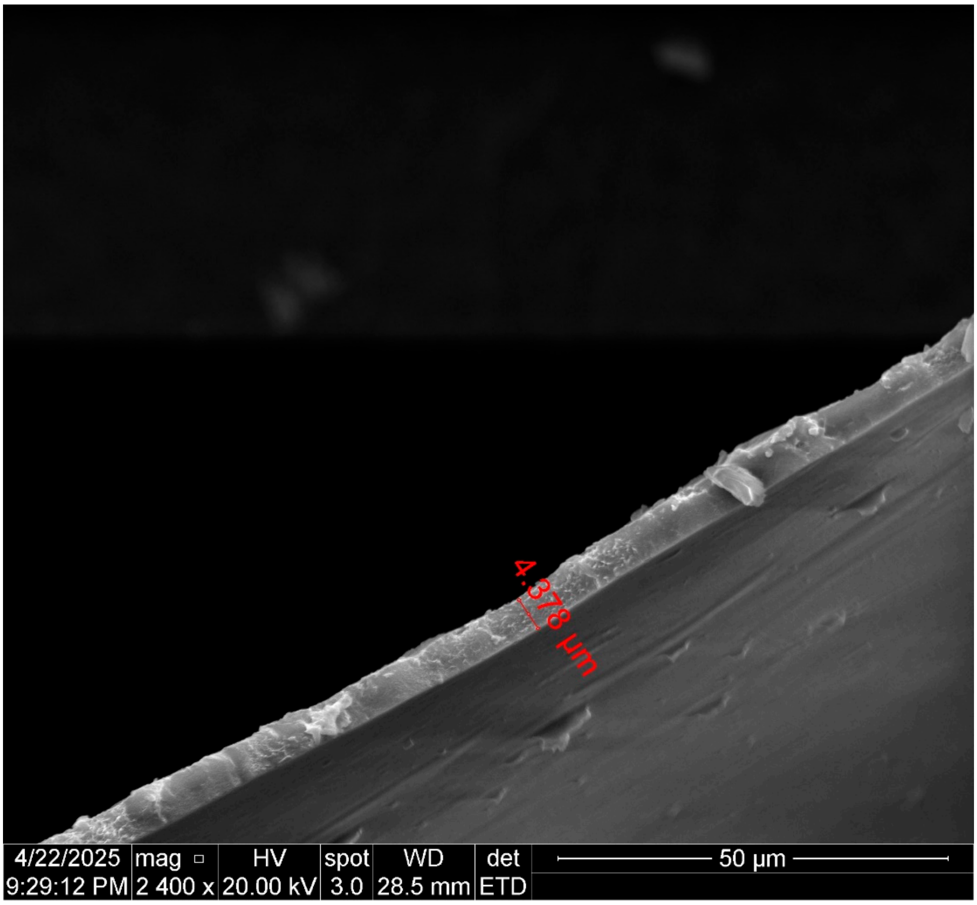

Figure S7. Cross-sectional structure of the **TPTc-DBD/PVDF** composite film.

The thin film thickness test was conducted as follows: the prepared thin film was placed in liquid nitrogen for 3 minutes, after this, it was brittle fractured. Then, the cross-section structure of composite film was observed via SEM. The result showed that the thickness of the TPTc-DBD/PVDF composite film was approximately 4.378 μm.

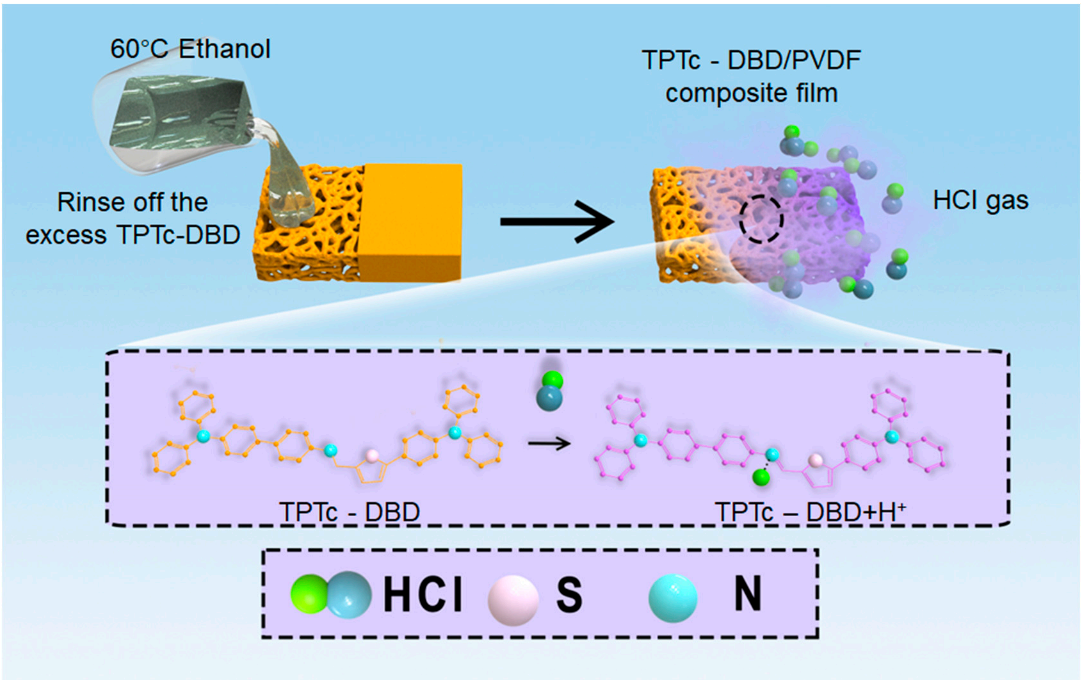

Figure S8. Schematic diagram of the thin composite film construction and its sensing mechanism to HCl.

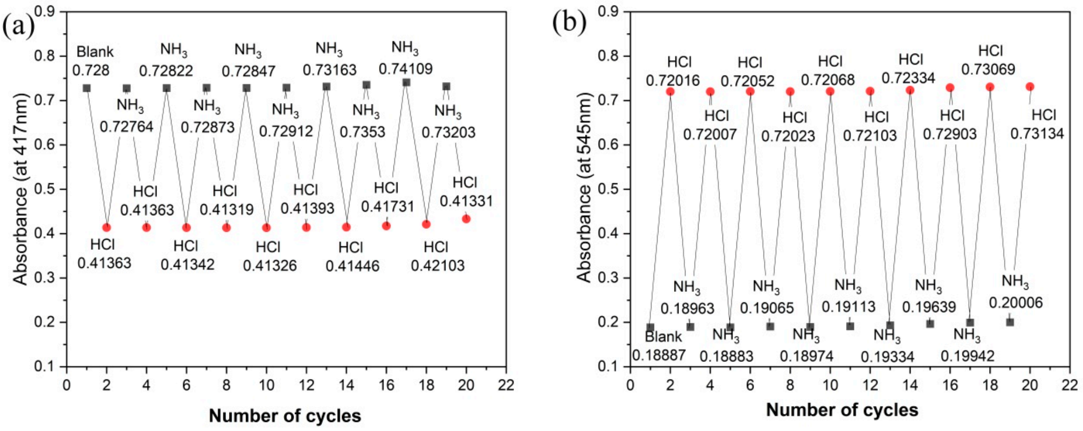

Figure S9. (**a**) Uv-Vis absorbance at 417 nm of TPTc-DBD/PVDF composite films in HCl and NH3 atmospheric environments during ten test cycles, (**b**) Uv-Vis absorbance at 545 nm of TPTc-DBD/PVDF composite films in HCl and NH3 atmospheric environments during ten test cycles.

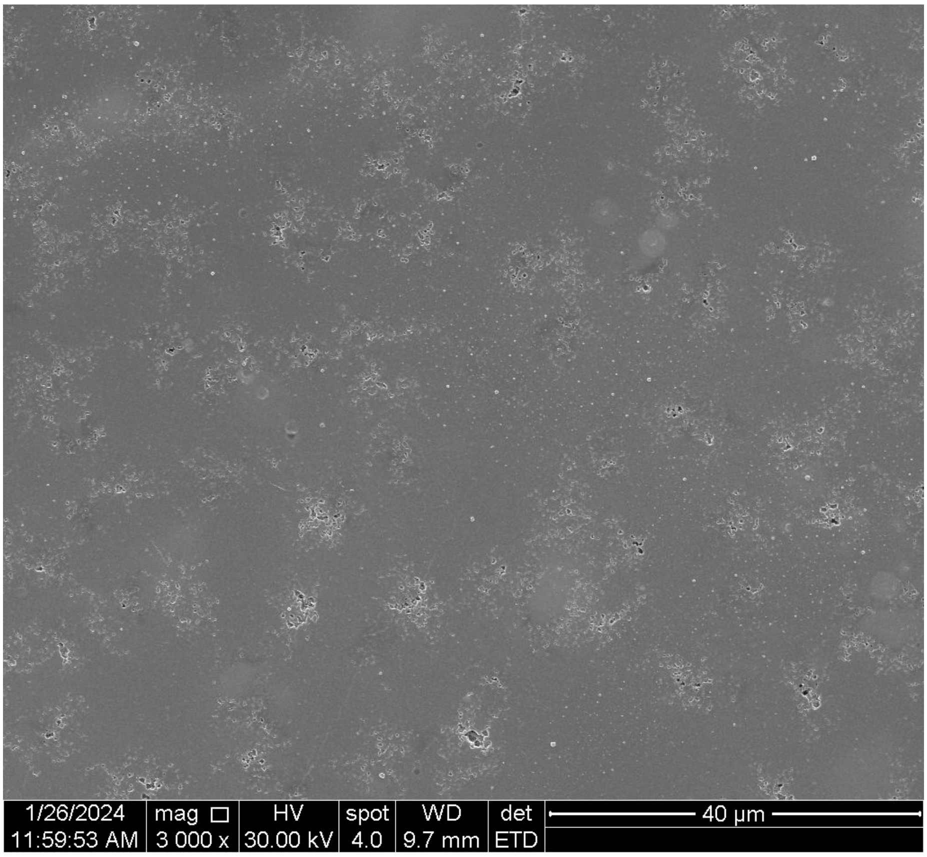

Figure S10. The morphology of TPTc-DBD/PVDF composite film after ten test cycles.

Table S2. A comparison of sensing performance on HCl between some previously reported sensors and the sensor in this work.

References	Article title	Publication time	Sensing group	Detection method	Detection method
[31]	Optochemical sensing of hydrogen chloride gas using meso-tetramesitylporphyrin deposited glass plate	2008	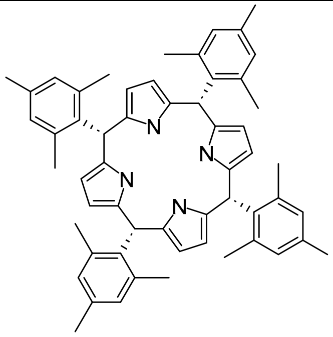	Absorption spectrum	0.1 ppm
[32]	Nanofiber-net-binary structured membranes for highly sensitive detection of trace HCl gas	2012	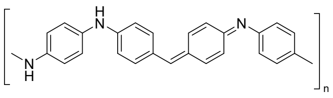	Absorption spectrum	7ppb
[33]	Schiff-base as highly sensitive and reversible chemosensors for HCl gas	2013	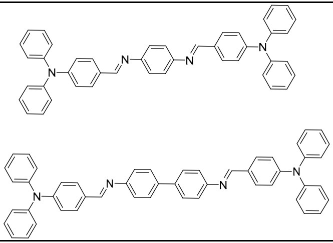	Emission spectrum	0.03ml
[34]	A triphenylamine-based benzoxazole derivative as a high-contrast piezofluorochromic material induced by protonation†	2014	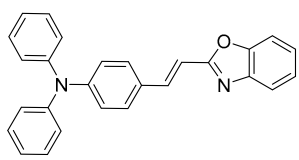	Absorption spectrum	There is a color change
[35]	Dye Modifi cation of Nanofi brous Silicon Oxide Membranes for Colorimetric HCl and NH 3 Sensing	2016	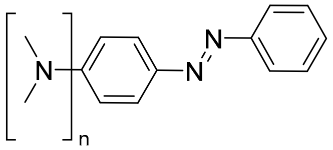	Absorption spectrum	100 ppm
[36]	Porphyrin-Functionalized Hierarchical Porous Silica Nanofiber Membrane for Rapid HCl Gas Detection	2018	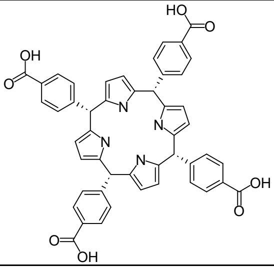	Absorption spectrum	17 ppb
[37]	Polyaniline-Functionalized Nanofibers for Colorimetric Detection of HCl Vapor	2018	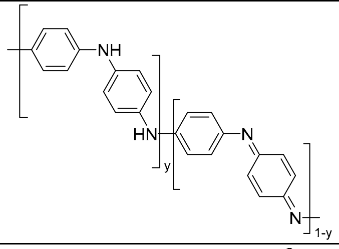	Emission spectrum	80 ppb
[38]	Aggregation induced emission enhancement -active triarylamine-based polyamides containing fused ring groups towards electrochromic smart	2021	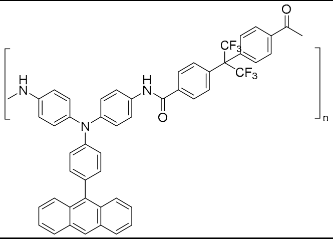	Emission spectrum	There is a color change

	window and sensor for HCl and TNP				
[39]	Smart Metal–Organic Frameworks with Reversible Luminescence/Magnetic Switch Behavior for HCl Vapor Detection	2021	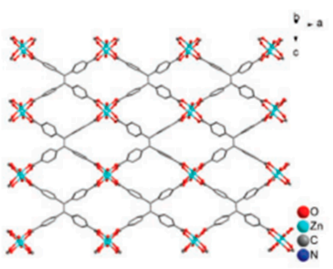	Emission spectrum	2.63 ppm
[40]	Zn(II) Complexes Based on a Schiff Base: Mechanochromism- and Solvent Molecule-Dependent Acidochromism	2022	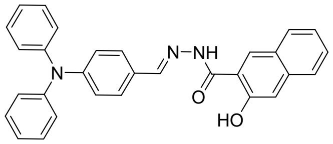	Absorption spectrum	There is a color change
[41]	Imine Bond-Based Fluorescent Nanofilms toward High-Performance Detection and Efficient Removal of HCl and NH3	2023	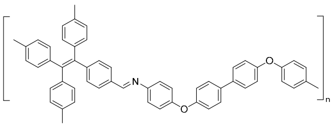	Emission spectrum	150 ppb
-	This work	2025	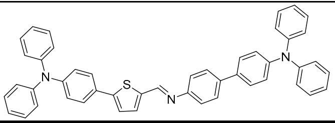	Absorption spectrum	3.56 ppm
